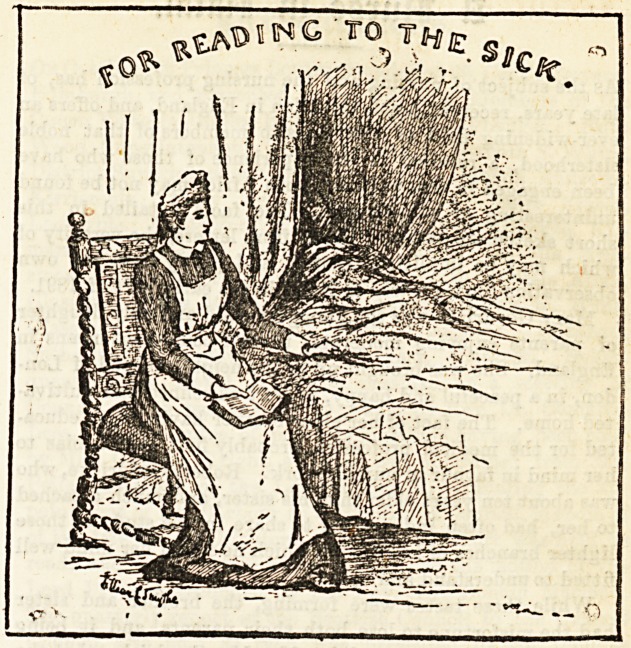# The Hospital Nursing Supplement

**Published:** 1891-10-24

**Authors:** 


### "The Hospital" Nursing Mirror.

Being the Extra Nursing Supplement of "The Hospital" Newspaper.

Contributions for this Supplement should be addressed to the Editor, The Hospital, 140, Strand, London, W.C., and should have the word "Nursing" plainly written in left-hand top corner of the envelope.

### En Passant.

Nurses in Belvedere.—For some time the Committee of the Belvedere Fever Hospital at Glasgow have been considering how to reduce the nurses' hours; and this is now to be done from fourteen and fifteen hours for day and night nurses to ten and eleven respectively. Also the Committee propose to increase the nurses' wages every year of service by 5s. a month, so that a nurse of ten years' standing will receive £45 a year. The alterations will necessitate an increase of the staff, and consequent building of more nurses' rooms, but we are sure the ratepayers will not object when the reform is so obviously needed.

Nurses for the Middle Classes.—Another of our correspondents writes to say that as she is a private nurse working on her own account, she is ready to go to middle-class patients for 10s. or 15s. a week, and do "whatsoever her hand findeth to do." She sends her address; this makes the second nurse who has expressed her willingness to meet what is undeniably a great want. We advise our two correspondents to let the doctors near them know of their readiness to take such cases. Of course it would be a great and a good work to organise an agency for bringing the want and the workers together, if there were only someone ready to and capable of doing it.

Nurses' Instruments.—Turning over Bailey and Son's catalogue of appliances one learns how, where expense is no object, nursing can be made easy. Here we see figured electric call bells, elaborate bed tables with book rests, all sorts of cunningly contrived air and water cushions, hot-water bottles, and bandages and dressings of the most accommodating order. Here also we learn that crutches, water-beds, &c., can be had on hire; a point which private nurses may be glad to know. Towards the end of the catalogue are illustrations of chatelaines, wallets, and cases for holding the few instruments every nurse carries. Chatelaines jingle and the wallets are far preferable. Elbow-scissors so useful for cutting off bandages and dressings; the American spatula, rough edged, and with a hole in the middle; half-minute thermometers, poultice knives, bandage-winders, feeding cups, and even safety pins are here illustrated, and a nurse can gain many hints from running her eye over this catalogue.

Short Items.—A Registry of Cottage Nurses, in connection with Miss Broadwood's scheme, has been started at Grange House, Cambridge.—Mrs. Lionel Lucas has offered to establish a Jewish Home for Nurses at the East-end, if the Jewish Board of Guardians will contribute £100 a year towards the expenditure.—The jury, after the inquest on Jeanette A. Ferrier returned a verdict of death from valvular disease of the heart, accelerated by exhaustion and exposure; a verdict which covers a sad story and a serious warning to all nurses to provide for their old age.—Mrs. Brewer has gone abroad, and hopes to visit the chief Italian hospitals.—Will our readers please remember that we are always glad to receive news of appointments, presentations, or any matters of interest?—There was a large audience at the Nurses' Club last week when Mr. Gustavus Hartridge gave a most interesting lecture on "The Eye," illustrated by numerous diagrams and instruments.—The Conversazione of the Royal British Nurses' Association will be held on December 4th.—Miss Gordon, of the London School of Medicine for Women, has been appointed Assistant Medical Officer at the Shadwell Children's Hospital.—The Local Government Board has again demanded Dr. Collie's resignation, pointing out that the administration of the hospital and the tone of the nursing staff had been unsatisfactory under his rule.

Improvements at Norwich.—The following recommendations have been submitted to the Governors of the Norfolk and Norwich Hospital by the Board of Management:—

1. That probationers be relieved of much of their scullery work, and allowed from the first to assist in the ward to which they are attached, and occasionally in the casualty department, in the way of nursing and surgical dressing, under proper superintendence.
2. That probationers be not entrusted with night duty until six months' training.
3. That each night nurse have charge of one ward only, and not of two as at present; and that she be relieved of attendance at the patients' dinner hour.
4. That a system of night superintendence should be instituted.
5. That the wardmaids be required to relieve probationers and nurses of much of the scullery work now done by them.
6. That nurses shall not as a rule be required to undertake private nursing during their period of training.
7. That probationers and assistant nurses be instructed in their duties by the Lady Superintendent (See Law 89), and by the head nurses, with special reference to the lectures given by the medical staff.
8. That day nurses be not called upon for night work after day duty, except in cases of special emergency, and that no nurse commence her ward work before breakfasting.
9. That certificated nurses only be appointed Sisters or head nurses.

Norwich is a beautiful hospital under excellent management and with all the latest improvements. If it passes the above recommendations it will almost attain perfection.

Attendants in Trouble.—The necessity of improving the *personnel* of attendants as far as possible is exemplified by a sad occurence at Sunnyside Asylum, which is thus reported by the medical officer:—

"The register of accidents contains three entries. One refers to a severe scalp wound self-inflicted, one to fracture of the radius due to a fall, and one to bruising of the hand and back. The case of bruising the hand and back refers to the case of a woman who was subjected to ill-usage, believed to have been inflicted by one or more of the attendants. The patient, who was suffering from acute mania and had been very excited and noisy, was found to bear marks of bruises which, in the opinion of Dr. Havelock, acting for Dr. Howden, then absent on holiday, could not have been self-inflicted. He arrived at the conclusion after careful inquiry that the patient had been beaten by one or more of the attendants during the night, and in accordance with the regulations he immediately reported the circumstances to the General Board of Lunacy and to the Procurator-Fiscal of the County. As the result of the Procurator-Fiscal's inquiry three of the female attendants were tried for assault before the Sheriff, but he found the charge not proven. An additional inquiry was then made by Dr. Howden and Mr. Lyell, Clerk to the Managers, with the result of confirming the opinion arrived at by Dr. Havelock, and in consequence one of the attendants has been dismissed as guilty of rough usage, and other two have been discharged with a month's notice as having been more or less connected with the rough usage. Such ill-usage as appears to have been applied in this case cannot be dealt with too strictly and thoroughly. The Asylum authorities seem, however, to have done everything that the occasion required both in investigating and reporting the occurence, and in regard to taking steps to obtain the punishment of those who were implicated."

The name of the dismissed attendant is Isabella Clark. Scotch asylums have a good reputation, so that this scandal was most unexpected, and is a great grief to the authorities and to the better class of attendants.

### Lectures on Surgical Ward Work and Nursing.

By Alexander Miles, M.D. (Edin.), F.R.C.S.E.

### Lecture XXXVIII.—Some Instruments Used in Connection with Operations on Bone.

(1.) Probes.—These are used for very various purposes in surgery, chiefly, however, to explore sinuses in order to detect their extent, direction, and contents. They should only be employed when the finger of the surgeon is not available, either on account of the small size or great depth of the sinus, as no information derived by the use of the probe is to be compared in value to that given by "the educated finger." This applies especially to such conditions as scalp wounds where it is of great importance to ascertain the condition of the bone, and where the wound in the soft tissues may sometimes be enlarged rather than trust to the probe as a diagnostic agent. The ordinary short silver probe supplied in every pocket case is the type of this instrument. It is about six inches long, rounded, with a slightly bulbous point at one end, and at the other flattened out and furnished with a large needle-eye. Being of silver, it may be made to take any shape desired.

The *gun-shot probe*, as its name implies, is used to follow up the track of a bullet. It is about twice as long and as thick as the smaller probe, but otherwise identical with it.

For the same purpose Nélaton introduced his ***bullet probe***, which is furnished with a small porcelain head, so that when it touches the bullet of which it is in search a black mark is left by the lead on the probe, and thus any source of error such as might arise by touching bone or other hard substance is eliminated.

*Spiral probes*, made of a fine continuous steel thread, have been used to follow up the sinuous track of a bullet, but their utility is limited, and the difficulty of keeping them aseptic is a great disadvantage.

*Periosteum Separators or Elevators* are very often employed in operating on diseased bone, their use being indicated by their name. They are of very various shapes, but all agree in having a blunt edge and a strong handle, the former to peel off the periosteum, the latter to give the necessary leverage. The pattern used by Dr. McEwen, of Glasgow, is of very general utility, affording great leverage, and taking up a small amount of room.

### Forceps used in Operations on Bones.

(*a*) *Necrosis or Sequestrum Forceps.*—These are used to extract pieces of dead bone—sequestra—from a wound or sinus.

They have an ordinary "scissors joint," have rough gripping points, and require to be of considerable strength. They may be straight, or curved.

(*b*) *Lion Forceps*, originally used by Sir William Fergusson in excising the upper jaw, may be employed in almost any condition in which it is necessary to get a very firm grip of a piece of bone. The blades beyond the joint are more curved than those of necrosis forcep, and each terminates in four strong teeth, those of the two blades being opposed.

(*c*) *Gouge Forceps.*—These are used to remove fragments of bone piecemeal, rather than complete sequestra. They practically consist of two gouges united so as to form forceps, and are exceedingly powerful. The edges are sharp, so that even hard bone may be readily pared down by them. Like others, they are made curved as well as straight.

(*d*) *Bone Forceps*, or *Bone Pliers*, as they are sometimes called, are cutting instruments, and are used under various conditions, taking the place of the saw in small amputations, such as fingers or metacarpals, or in cases other than amputation, where bones require division.

*Chisels* are used either to chip away portions of bone, or to divide a bone completely through, the size and shape of the instrument varying, of course, according to the purpose for which it is employed.

The most powerful and generally useful form of chisel is that introduced by Dr. McEwen, of Glasgow, for his operation of osteotomy, and hence sometimes called "McEwen's Osteotome." The whole instrument, blade and handle, is of one piece of metal; the blade is bevelled equally on both sides, wedge-shaped, and must be of the best steel. It is graduated on the side to ensure accuracy in dividing the bone.

Other forms of chisel have handles of wood, ebony, and so on.

The illustrations are used by kind permission of Messrs. Maw, Son, and Thompson.

### Appointments.

Charing Cross Hospital.—Miss Duff, late lady pupil, has been appointed Night Superintendent at Charing Cross in place of Miss Amy Johnstone, who has gone as Matron to Boston Hospital.

Paddington Infirmary.—Miss Annie Bragshay, of St. Mary's Hospital, Paddington, has been appointed Assistant-Matron of this infirmary.

West Herts Infirmary.—Miss Catherine Wilkinson has been appointed Matron of this infirmary at Hemel Hempstead. Miss Wilkinson trained six months at Aberdeen and two years at Wakefield. In 1888 she went to Derby as assistant Matron, finally becoming Matron. Owing to the outbreak of illness amongst the nurses, and the state of transition of the Derby Infirmary, Miss Wilkinson has had a hard time lately, and we hope a lighter and more congenial post awaits her at West Herts.

Fleming Memorial Hospital.—Miss Ellen Daintree, at present Assistant Matron at Worcester, has been appointed Lady Superintendent of the Hospital for Sick Children, Moor Edge. Miss Daintree was for five and a half years at Great Ormond Street, three years at Bradford, and eighteen months at Newark. Her testimonials are excellent. Miss Annie Mulligan, who trained at Sir Patrick Dun's Hospital, and had about eight years' experience there, has been appointed Sister at the Fleming Memorial.

### Help for the Dying.

The sale in aid of "Friedenheim" recalls one of London's great needs—more homes for the dying and more careful nursing for them. In the hurry of our great hospitals there is no room for those worsted utterly in the battle of life; if there is any hope, then the doctors accept the case and fight inch by inch against the enemy death; but if the case is hopeless—if it is only peace and tendance through the drear valley of the shadow that the patient seeks, he must go elsewhere: back to his own home, if he has one, if not, to the workhouse infirmary. At the workhouse he takes his chance of skilled attendance; there may be deft and trained nurses there to wait on him; but again, there may not. In vain does the Workhouse Infirmary Nursing Association plead for more funds and more probationers; the public are not interested in sick paupers, they prefer to give their guineas to more sensational charities. Yet Miss Gill, the Assistant Secretary, writes: "We have far more applications for nurses than we can possibly fill owing to the want of funds. We can only train a certain number, and it is often difficult to get outside nurses at the moment one wants them. At the present time all our probationers in training are engaged as fast as they complete their training." Failing the workhouse, we know of only two refuges for the dying—Friedenheim, and the Catholic Hospital of St. John and St. Elizabeth in Great Ormond Street. And even the latter is sadly crippled for want of funds. One strolls into its wards and wonders at the poverty-stricken look, the faded paint, the dusky ceilings, so different to the smartness of other hospitals. The Reverend Mother pleads for even the smallest subscription from the visitor, and one cannot choose but give. Truly, the dying here have one comfort not often provided—every ward opens into the adjoining chapel, and during service the odour of the incense and the sweet tones of the chanting sounds solemnly through the wards. This is far better than any music the Guild of St. Cecilia can supply. If any of our Catholic readers do not know this hospital, and its needs, we beg them to visit it and subscribe to it.

Dublin has a model hospice for the dying, founded by the Irish Sisters of Charity, in a large old house called Harold's Cross. It was opened on December 9th, 1879, and now contains 180 beds, and is worked by 30 Sisters, headed by Mother Mary John. Madame Belloc (*née* Bessie Parkes) lately wrote a beautiful description of this hospital, and expressed her desire to get a similar institution established near London. But Cardinal Manning points silently to the poverty-stricken hospital of St. Elizabeth, and bids Catholics place it in a firm financial position 'ere they give their alms elsewhere.

We fall sick and get well again, and remembering our pains and how they were made bearable by the skill of surgeon and nurse, we send a handsome donation to some hospital. But the dying do not get well again; however much they may have appreciated the peace secured to them in their last moments by tender care, they cannot express it. Silence falls on us as we enter the valley of the shadow. But is it not, therefore, all the more the duty of the rich relatives of the dying to grant to the poor, who would otherwise lack it, a place of peace and comfort during their last few days of life?

### A Bed for a Sick Nurse.

We have received the following subscriptions towards endowing a bed for a sick nurse at the Brassey Home, between October 13th and October 20th. One subscription has come to us from America with a charming letter of sympathy. Another is from a gentleman who writes "you ought to have no difficulty in getting thirty subscribers from amongst those who, like myself, owe so much to skilled nursing." Miss James of Philadelphia, £1 1s., Mr. Thomas H. Nevill, £1 1s., Mrs. Creighton Hale, £1 1s.; Miss Berry, £1 1s.; Nurse Mary Hays, 1s.; Nurse E. M. Axton, 6d.; Miss Wilson, 5s.; One of the Second Thousand, 6d., ; A. J. R. 6d.; One of the First Thousand (Hampstead), 2s.; and Nurse Stockwell, 2s. 6d. This makes eleven guinea subscriptions and £1 4s. 6d. in smaller sums, leaving eighteen more guineas still needed.

### TRIFLES.

Strange as it may seem, trifles are often far harder to bear than great trials. For instance, we have had an accident or an illness which, though not fatal, threatens to injure us for life. When the certainty is first borne in on us we are overwhelmed, and feel we shall never be able to mix with our fellow-creatures again, or show our maimed bodies or scarred faces among our mates. Yet as time passes on we grow accustomed to the thought, and the kindness of friends and the love of our relations are so consoling, that almost, in spite of ourselves, we are comforted, and begin to think we are heroes to take God's chastenings so well.

Unfortunately, this exaltation of spirit is not lasting. Little worries, little vexations crop up; they are "the little foxes which spoil our grapes." People have ceased paying us so much attention, and our attendants have other calls on their time, so we think ourselves neglected. Then noises jar on our weakened nerves, we crave for food that would hurt us. we cannot move about, and feel like birds shut up in a cage, and are tempted to beat our wings against the bars; let us only stay quietly on the perch, dear friends, and we shall not feel the bondage and imprisonment.

The homely proverb, "None can tell where the shoe pinches but he who wears it," is a very true saying; perhaps if our trials could be seen and known our friends would try to remove them. In the meantime, as they cannot, these worries produce so much vexation and raise up so many evil thoughts, wishes, and desires in our hearts, without any adequate good effects, that they appear quite unnecessary to us. Yet if these trials can produce so much evil, why cannot they produce an equal measure of good? It has been well said that "Their very character of trifles it is that makes them useful; they try us secretly, insignificantly, and yet sharply. Think of the crown of thorns borne for you. Did that cause no suffering? yet what are thorns?"

Such experiences have been the lot of hundreds because our Master who was made perfect through suffering would have us like Himself. These trials if taken as from God's hand will make us patient; they will teach us to control ourselves, to be thoughtful and considerate to all about us. What weak, feeble-minded people we should be with nothing to try us. Let us face things boldly in Christ's strength, and our crosses will blossom, our crown of thorns turn into one of roses. We will pray with David, "Lord, make Thou all my bed in my sickness;" then with His watchful care to help us the "trifles" will get smaller and smaller till they will disappear entirely, and we shall wonder how we allowed our minds to be disturbed by "trifles light as air."

### A Nurse in Natal.

As the subject of nursing and the nursing profession has, of late years, received much attention in England, and offers an ever-widening field of usefulness to members of that noble sisterhood, a relation of the experience of those who have been engaged in this work in South Africa may not be found uninteresting or unprofitable. The facts detailed in this short sketch are gathered partly from letters, the veracity of which may be implicitly trusted, and partly from my own observation during a trip to Natal in the early part of 1891.

Mary Goodricke was the youngest child and only daughter of parents enjoying moderate, though competent means in England. She was brought up in the neighbourhood of London, in a peaceful and happy, as well as refined and cultivated home. The fact of her elder brother having been educated for the medical profession probably first gave a bias to her mind in favour of nursing work. Robert Goodricke, who was about ten years older than his sister, and much attached to her, had often induced her to share in the study of those lighter branches of knowledge which he found her mind well fitted to understand and value.

While these tastes were forming, the brother and sister had the misfortune to lose both their parents, and it being discovered, on the death of the elder Mr. Goodricke, that the greater part of his estate had been swamped in unsuccessful speculations, the young people found themselves dependent almost entirely on their own exertions. Dr. Goodricke had, as yet, but a very small practice in London, and it was now that his sister determined to adopt the nurses' profession, both as a means of self-support, and also as an outlet for those tastes and aspirations which, in her, took the place of the accomplishments natural to other women. Her brother's influence easily secured her entrance as a probationer in the London Hospital. Here she devoted herself to becoming a thoroughly accomplished and efficient nurse; taking advantage not only of the excellent practical training provided in that "School for Nurses," but also of the admirable lectures delivered yearly to probationers on the plan adopted by Dr. Allchin and Miss Lückes.

As she advanced through the course, her natural and instinctive interest in the subject coming, no doubt, to her aid, every faculty of Miss Goodricke's mind became more and more absorbed in her work.

The great and almost sacred importance of her calling revealed itself to her as the months rolled on. She recognised the vast and almost limitless influence for good which a faithful, earnest nurse possesses; and strained every energy to reach the high standard thus set before her, taking, as her motto, the admirable words of Sir Frederick Leighton: "Whatever of dignity, whatever of strength we have within us, will dignify and make strong the labours of our hands; whatever littleness degrades our spirit, will lessen them and drag them down. Whatever noble fire is in our hearts will burn also in our work; whatever purity is ours will chasten and exalt it; for as we are, so our work is; and what we sow in our lives, that beyond a doubt we shall reap, for good or for ill, in the strengthening or defacing of whatever gifts have fallen to our lot."

This self-concentration to the attainment of a lofty ideal brought its own reward, and at the end of her year's probation Miss Goodricke, having passed her examination successfully and obtained a certificate, was admitted by doctors, Matron, and Sisters to be one of the most trustworthy and efficient nurses in the hospital. At the commencement of the third year she was elected to the rank of Sister, and shortly after this her brother received an invitation from an old college friend, who had been a few years settled in Natal, and had a good practice in Durban, to join him there as partner and assistant. This offered so good an opening to the young doctor that both brother and sister felt it must not be refused, though the parting which quickly followed was very painful to each.

Dr. Goodricke had been about six months in his new sphere, and Mary's third year of hospital work was nearly completed, when a letter from him reached her urgently requesting her to come out to him. "Though doing well here," he wrote, "and meeting with much kindness, it does make one feel lonely at times to be so far from home and from all one's own people; and as you and I, Mary, are the only ones left, it seems hard that we should be so widely separated. Therefore, make no fresh plans in England, but come to me as soon as you can arrange matters after leaving the hospital.

"I know nothing would induce you to give up your work, but trained nurses are badly needed here, and I think I can promise you sufficient employment amongst my own patients alone. Of course, you will have to make up your mind to find things different here in many ways from what they are in England; but that is only to be expected, and I am vain enough to think you will willingly put up with a few colonial inconveniences for the pleasure of being near me." He then went on to say that he wished her to persuade an elderly aunt, their sole surviving relative, with whom he had lived since his parents' death, to accompany Mary to Natal.

This would be protection for her during the voyage, and he thought Mrs. Thornton would be happier herself and make them happier by sharing their new home than by remaining alone in England.

When Mary first mentioned this project to the aunt, Mrs. Thornton would not hear of it. She was no longer young, and was naturally very conservative in her tastes and habits. All her life had been spent in England, chiefly in London, and she could not imagine an existence apart from all the familiar surroundings of sixty-five years.

"Do not talk of it, Mary," she said, decidedly, "it is impossible. It may be your duty to go to your brother; but though it will break my heart to lose you, I am sure it would kill me outright to give up my home and cross the sea. I could never do it."

"I know it would be hard for you, Aunty; but when the wrench was once over, think how happy it would make you to be always with us, and how proud you would be of keeping house for Robert. You know he always did need you to look after his little comforts, and I am so stupid at that kind of thing. Then think how dreadful it would be to be quite separated from us! You would be quite lost with no Mary to visit and tease you." By the constant bringing to bear of arguments such as these, Mrs. Thornton was at last induced to give a reluctant consent.

(*To be continued.*)

### Glasgow Grumblers.

The Committee which has been enquiring into the complaints of the nurses of the Glasgow Royal Infirmary issued their report on Tuesday. They first explain that they have examined twenty-three nurses, the house physician, two of the visiting staff, and the superintendents, and the cook. With regard to the complaints about the food, the Committee pronounce them, if not wholly without foundation, at least much exaggerated, and they merely recommend the Matron to secure more variety, and that the cook be supplied with an extra assistant. With regard to the long hours, the Committee recommend that the monthly holiday be a regular institution, and that the lecturer be asked to deliver his lectures in the evening, so that the night nurses should not have to rise early on these occasions. Practically, the verdict is against the complainants, but we will give the full report next week.

### Everybody's Opinion.

*[Correspondence on all subjects is invited, but we cannot in any way be responsible for the opinions expressed by our correspondents. No communications can be entertained if the name and address of the correspondent is not given, or unless one side of the paper only be written on.]*

### SIX MONTHS' CERTIFICATES.

"S. G. M." writes:—In corroboration of Miss Morant's statement, I should like to mention that last year, while nursing a relation who had an attack of typhoid fever, I sent for a private nurse from a nursing institution in the South of England. On her arrival I enquired what training she had had. She told me that she had been for six months to the Middlesex Hospital. During that time she had not had an opportunity of nursing a case of typhoid fever, but she said she had had it herself, and so knew what ought to be done! I must add that the Institution charged two guineas a week for the services of this nurse. I am glad you have called attention to this system of sending out half-trained probationers as competent nurses.

"An Old Middlesex Nurse" writes: Many will be glad to see in your issue of last week the protest you make against the granting of certificates by the Middlesex Hospital to its paying pupils qualifying them as trained nurses after six months' attendance at the hospital. I know of two cases out of many at the Middlesex Hospital, in which nurses, after, in the one case, three, and in the other five years' service, have been refused the certificate, or granted only a modified one for some trifling error. Yet it is hardly possible that these can have learnt less or gained less experience than those who have attended only six months. These latter, it is true, have paid a fee, but only, it would seem, to excuse their learning that very necessary portion of their duty, the attendance to the general comfort of their patients. Such conduct brings disgrace on nurses as a body, not only on the half-trained ladies of the Middlesex, for many people do not wait to ask whether the nurse they hire trained for a half-year's half-service and paid a fee, or whether she trained for three years' full service. And the ignorance and airs of the first are accredited to nurses as a whole, hence we constantly hear that private nurses are more a bother than a blessing.

### NURSES AS PATIENTS.

"Nurse L." writes: I have just read the letter written by "S." in The Hospital of last week. Like "S." I was obliged to undergo an operation, and was a patient for four weeks in a general hospital, and I, too, am a nurse of some experience; but there the likeness between our two cases ends. I do not, of course, know what hospital "S." was in. I was a patient in the London Hospital, and I can only say that I was treated with the greatest kindness and consideration by doctors, Sisters, and nurses, the Sister and nurses of our ward in particular often giving themselves unnecessary trouble to do many things which they thought would add to my comfort. As that was my experience, it is only fair and right I should give my testimony on behalf of *some* nurses in our hospitals.

### JAMES PAYN'S NURSES.

"Rosa" writes: The quotations from James Payn's book you give might be facts. When I first nursed among the sick poor I came across just such things, told me by the village nurse, done not to get the "job" over, as he says, but in all kindness—according to their idea of kindness—nay, they used the very words. Speaking of one who had a severe struggle towards the end, the nurse said she just put her hand over his mouth and held his nose, and he went off like a lamb, poor dear. And I believe it was often done if they "died hard," as they called it. As for poor illegitimate children (always insured, by the way), I have seen them washed by being held under the cold water tap. This was 25 years ago in a large village. A man went to one institution only the other day, asking to see his brother "before he was smothered," as he heard he was very ill. So it may be done among the ignorant still, who think it a kindness to release patients from their suffering as quickly as possible.

### ALL SAINTS' NURSES.

Eight Nurses write: In this week's Hospital there is a notice, "The private nurses of University now receive an extra 5s. a week while they are at cases. Considering how low their salaries are, this is a step in the right direction." As old nurses of this Institution we must beg to correct this statement. First of all, there are no private nurses of University, as those nurses belong to the All Saints' Nursing Home, Fitzroy Street, although we receive our training at the University, and care loyally for the Hospital. We receive very good wages, and, in addition to our wages and uniform, we have a percentage of 2s. 6d. in the pound on every case we nurse, the percentage in some cases amounting to 10s. 6d. a week. If we are incapacitated by illness or old age we receive a pension, and are provided for at the Home or elsewhere. If we require a long rest we receive "full pay" for three months, and after that time "half pay" for a second period of three months, and are provided for either at the Home or the Cottage. We have a Cottage at Eastbourne, where, in cases of illness or over-fatigue we go free of expense; and if we wish to spend our holiday there, we can do so by paying 5s. a week. This Cottage is made as home-like as possible, and we all appreciate it. Much is said in these days about nurses taking their own earnings, but those who advocate this do not remember that slack times and times of illness come to nurses as well as to everyone else, and then all goes out and nothing comes in; and there are many expenses and anxieties which we are saved who keep steadily on with friends who care for us and our interests, and do not only pay us when we are earning.

### Presentations.

On October 6th Princess Louise attended at 29, Castle Terrace, Edinburgh, and presented to the following Members of the Queen Victoria Jubilee Institute their official badges: Ruth Wood, Superintendent, Glasgow Home; Elizabeth S. White, District Superintendent, Glasgow Home; Isabella Andrews, Kirriemuir; Mary Armstrong, Montrose; Eliza Fraser, Lockerbie; Annie Ford, Kirkcaldy; Mary Monkhouse, Kilmarnock; Margaret Tetham, Kilmarnock; Isabella Armstrong, Edinburgh; Ellen Wilcox, Leith; Jessie Allan, Glasgow; Alice Maria Epps, Glasgow; Frances H. Hunt, Glasgow; Mary Elizabeth Neill, Glasgow; Elizabeth Sutherland, Glasgow; Annie Towers, Glasgow. Subsequently Her Royal Highness presided at a meeting of Council, which was opened by prayer by Rev. Dr. Cameron Lees. The minutes of previous meetings having been read by Miss Guthrie Wright, a letter was submitted from Mr. Peile, President of the London Council, intimating the appointment of Dr. Barbour as a Member of Council, in room of of the late Sheriff Crichton. The annual report was held as read, and its adoption was agreed to, on the motion of the Lord Provost, seconded by the Rev. Dr. Donald Macleod. The quarterly report of the Scottish Council to the Central Council, also held as read, was presented by Sir Douglas Maclagan, and its adoption was moved by Lady Campbell, seconded by Miss Lumsden, Aberdeen, and agreed to. Mr. Hotson gave in a satisfactory financial statement, and reported that a gift of £500 had been received as the nucleus of a Pension Fund for Queen's Nurses in Scotland. It was proposed by Sir Thomas Clark, seconded by Mrs. Trayner, that Her Royal Highness, as President, should be asked by the Council to obtain Her Majesty's sanction for the establishment of a Pension Fund in connection with the Institute. This was agreed to, and the meeting ended. Under the guidance of Mrs. Ford, Miss Guthrie Wright, and others, Princess Louise later on went over the whole building and carefully examined its equipment, conversing freely with the nurses as she passed from room to room. The visit, with which her Royal Highness was apparently highly delighted, lasted nearly an hour. A very pretty picture of the presentation appeared in the *Daily Graphic*.

On October 14th, a pleasant gathering was held at the Royal Hants County Hospital, when the annual prizes and certificates were presented. The Chairman, Mr. Portal, presided, and said that the class of nurses consisted of thirteen or fourteen who had been through the examination, and it was exceedingly interesting to find how useful and varied their occupation and business seemed to be. He found that four were attached to wards in the hospital, three were on private nursing staff, two were nurses at the Public Hospital in Sheffield, and one was qualifying for India—and they heartily welcomed her presence that afternoon: he alluded to Miss Gaston—one was working for the Workhouse Nursing Association, and another under a Nursing Association in Cheltenham. This showed what wonderfully varied and splendid work was being done in that Hospital in connection with outlying districts and places. The prizes had been awarded after a good deal of thought, the name of the candidate not being attached to the competition paper, but a motto. Mrs. W. W. Portal then handed the prizes (consisting of valuable text books) to the successful competitors, viz.:—1st prize, Miss Mary F. Cosgrove; 2nd prize, Miss Ada Gaston; 3rd, for general proficiency, Miss Elizabeth Higgins. Each of the candidates who attended the course of lectures and examination was also presented with a certificate, which the Chairman had caused to be prepared as a memento.

### Wants and Workers.

*St. Andrew's, Stockwell.*—Jumble sales are held every three months for the benefit of the poor of this parish, and any of the following articles will be most thankfully received: old clothing of every kind, baby linen, boots, pieces of carpet, muslin, curtains, &c., house linen, kitchenware, crockery, books, furniture—in fact anything. Please forward parcels to Mrs. H. O. Scott, St. Andrew's Mission House, Southesk Street, Stockwell. Carriage will be willingly paid on any parcels, and empties returned if desired.

### From Pharmacy to Farming.

(*Continued.*)

After tea Edward returned to the subject of his future career; but Uncle Joshua was callous.

"'Taint no mossel o' use, Master Edward," said he, "I've said my say. Stick to chemisting, and I don't know as I mightn't leave a corner o' my will for 'e; but go shiftin' and choppin' about from one thing to t'other, and I wipes my hands o' ye, and screws up my purse strings tight—so!"

When the twilight faded, Edward entertained his uncle with tales of robberies in London.

"You knows a deal o' thieves' ways, my lad," remarked Uncle Joshua, in wonderment.

"One has to be sharp-witted in town," replied Edward. "We have swindlers of all kinds in the shop at times. Are not you afraid of being robbed sometimes, uncle?"

"I don't give the notion much thought," said the miser, uneasily. "An' if burglars was to get in, they'd have all their work cut out to find my little bit o' money."

"Confess that the 'little bit' amounts to several hundreds," laughed Edward.

"I makes it a rule to say nothink about my savings," returned Joshua, sternly.

Then, as the tall clock in the kitchen chimed ten, the fellmonger rose, and shook hands with his nephew before going his nightly tour around the house. After locking all the doors, he lit a candle, and ascended to his bedroom. It was his custom to look at his hidden wealth daily; so, after locking the door, and listening intently for a moment, he pulled aside a tattered strip of carpet at the foot of the bed, took a screwdriver from a drawer, and began to loosen a screw in a floor plank. After drawing four screws, he gently lifted the board, and exposed an iron safe resting on a wide beam. The intense silence of a sultry summer night was over the land. The only sound was the *tick, tick* of the tall clock in the kitchen, and the chirping of crickets below. Kneeling, with the candle-light upon his face, Joshua thrust his hand amid the layers of bank notes. All was safe. He replaced the board, and put the screwdriver under his pillow, to remind him to drive the screws in in the morning. Two hours after getting between the sheets Joshua was awake and listening. He was normally a sound sleeper, but to-night an irrepressible suspicion kept his nerves in a state of high tension. Strive as he might, he could not banish the thought that an impecunious young man, who knew the ways of housebreakers, was under the same roof with his precious lucre. But about one he fell asleep, and snored softly.

Edward fared even worse at the hands of Morpheus. He lay in a mental conflict betwixt self-respect and worldly interest. Pride prompted him to return to London on the morrow, and quit his mercenary relative with gentlemanly disdain; while the thought of his insecure and struggling future suggested several desperate resources. Besides his despondent cogitations, the gnawings of a mouse behind the wainscot kept sleep away. It was still dark when he rose from the troubled pillow, and hurriedly donned his clothes. Fifteen minutes later he was walking down the garden with his trunk.

The mists of dawn were stealing up the hill from the moist meadows, and there was a grey rift in the eastern horizon. As he opened the garden gate noiselessly he fancied that he saw a dark figure crouching behind a lilac bush. But he could not feel assured that he had seen a human form when he had shut the gate. In his state of nervousness he attributed the apparition to an optical hallucination. Six hours after he was in London.

(*To be continued.*)

### Notes and Queries.

### Queries.

(5) *Home for Epileptics.*—A lady would be obliged if anyone would tell her where an incurable epileptic lad, aged 22, would be received?

(6) *Male Nurses.*—Wanted at once, the addresses of institutions (other than the Hamilton Association) that train male nurses.—*Quettah.*

(7) *Masseuses Abroad.*—Is there an opening for a certificated masseuse at Algiers, Cairo, Teneriffe, or the Riviera?—*Nurse G.*

### Answers.

*Sister Alice.*—Under such circumstances the nurse would, of course, not claim salary from the people who nursed her; why rather one would think they ought to be paid, for they did the work. Whether she is entitled to salary from her institution depends entirely upon its rules.

*Christmas Competitions.*—Socks received from Miss Lyon. Please address all parcels to Christmas Competition, The Hospital, 140, Strand, London, W.C.

*Miss F. L. Elms.*—Eighteen months ago you wrote proposing an Endowed Bed for Sick Nurses; we could not then start the scheme, but would be glad of your help now.

*A Doctor's Daughter.*—A hospital ward is not the place to make love in; only the strictest etiquette can make the position the doctor and nurse hold towards one another correct, and love making introduces at once the question of sex. We know of hospital marriages that have been successful, but still the idea is one to be discouraged.

*Matron.*—You do not send name and address. (1) In a cottage hospital of eight beds (but no private ward) I have no nurse, one servant at £15 a year, an occasional charwoman, to whom last year I paid in all £1 8s. 8d.; and a laundress two days a week, to whom I last year paid £6 18s. The laundress does not stay the whole day except when necessary. On an average I have four beds occupied.

*Nurse P.*—Central London Ophthalmic Hospital, in the Gray's Inn Road, W.C.; Royal Ophthalmic Hospital, Moorfields, E.C.; Royal Westminster Ophthalmic Hospital, West Strand, W.C.; Kent County Ophthalmic Hospital, Maidstone; Royal Eye Hospital, Manchester; Midland Eye Infirmary, Nottingham, and many others. See "Burdett's Hospital Annual."

*Toe.*—See articles on "Dispensing" in *Nursing Notes* for September and October. Price 2d. 12, Buckingham Street, Strand, W.C. Write to the Principal, 40, Charlotte Street, Portland Place, W., about lessons.

*Christmas Competitions.*—Socks received from Nurse M. Trump. Please address all parcels to 140, Strand, London.

(1) In this hospital of eight beds I have only one nurse (thoroughly trained) and one servant. Laundry work not done here. I will be pleased to give further information if enquirer will write to me. Sister Ada, Cottage Hospital, Stanmore.

(2) A handful of Calvert's Carbolized Tow made into pads for round the wrists will help to keep away fleas. A pad inside each arm of the nightdress has an excellent effect.—*E. C.*

### Amusements and Relaxation.

**SPECIAL NOTICE TO CORRESPONDENTS.**

**Fourth Quarterly Word Competition commenced October 3rd, ends December 26th, 1891.**

Competitors can enter for *all* quarterly competitions, but no competitor can take more than *one first prize* or *two* prizes of any kind during the year.

Proper names, abbreviations, foreign words, words of less than four letters, and repetitions are barred; plurals, and past and present participles of verbs, are allowed. Nuttall's *Standard* dictionary only to be used.

The word for dissection for this, the FOURTH week of the quarter, being

"PHEASANT."

| Names. | Oct. 15th. | Totals. | Names. | Oct. 15th. | Totals. |
|---|---|---|---|---|---|
| Lightowlers | 39 | 46 | Nurse J. S. | 45 | 51 |
| Bonne | 47 | 54 | Jenny Wren | 45 | 51 |
| Morico | 48 | 55 | Darlington | 42 | 48 |
| Robes | 29 | 36 | Nurse G. P. | 29 | 35 |
| Dulcamara | 39 | 46 | Hetty | — | — |
| Psyche | — | — | Janet | 30 | 33 |
| Agamemnon | 43 | 53 | Jackanapes | 27 | 27 |

### Notice to Correspondents.

Competitors are requested to notice that the New Quarterly Word Competition commenced October 3rd. We think many cannot have realized this, for we are sure they will be glad to employ their winter evenings trying to gain a prize in so easy a manner.

All letters referring to this page which do not arrive at **140, Strand, London, W.C.**, by the *first post on Thursdays*, and are not addressed PRIZE EDITOR, will in future be disqualified and disregarded.

N.B.—Each paper must be signed by the author with his or her real name and address. A *nom de plume* may be added if the writer does not desire to be referred to by us by his real name. In the case of all prize-winners, however, the real name and address will be published.

## Figures and Tables

**Figure f1:**
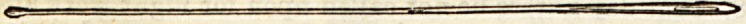


**Figure f2:**
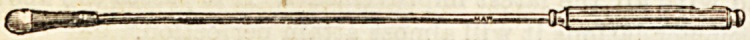


**Figure f3:**
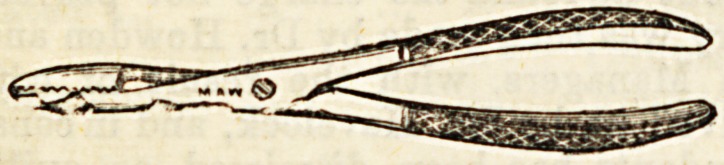


**Figure f4:**
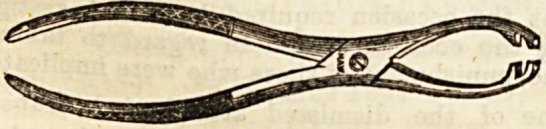


**Figure f5:**
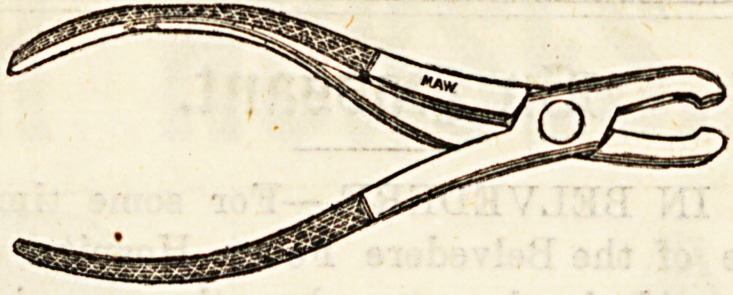


**Figure f6:**
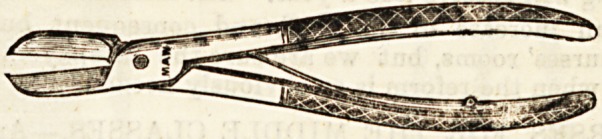


**Figure f7:**
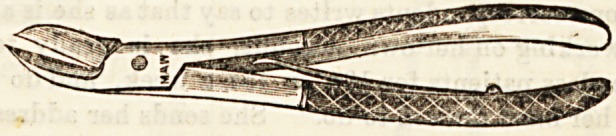


**Figure f8:**
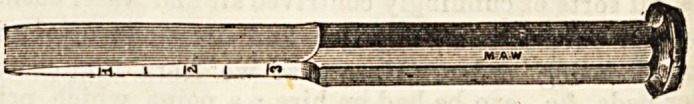


**Figure f9:**